# Homogeneous antibody–angiopep 2 conjugates for effective brain targeting†

**DOI:** 10.1039/d1ra08131d

**Published:** 2022-01-26

**Authors:** Yasuaki Anami, Wei Xiong, Aiko Yamaguchi, Chisato M. Yamazaki, Ningyan Zhang, Zhiqiang An, Kyoji Tsuchikama

**Affiliations:** Texas Therapeutics Institute, The Brown Foundation Institute of Molecular Medicine, McGovern Medical School, The University of Texas Health Center at Houston Houston Texas 77054 USA Kyoji.Tsuchikama@uth.tmc.edu Zhiqiang.An@uth.tmc.edu Ningyan.Zhang@uth.tmc.edu Chisato.Tsuchikama@uth.tmc.edu Aiko.Yamaguchi@uth.tmc.edu

## Abstract

Antibody-based therapy has shown great success in the treatment of many diseases, including cancers. While antibodies and antibody–drug conjugates (ADCs) have also been evaluated for central nervous system (CNS) disorders as well as brain tumors, their therapeutic efficacy can be substantially limited due to low permeability across the blood–brain barrier (BBB). Thus, improving BBB permeability of therapeutic antibodies is critical in establishing this drug class as a reliable clinical option for CNS diseases. Here, we report that, compared with a conventional heterogeneous conjugation, homogeneous conjugation of the synthetic BBB shuttle peptide angiopep-2 (Ang2) to a monoclonal antibody (mAb) provides improved binding affinity for brain microvascular endothelial cells *in vitro* and accumulation into normal brain tissues *in vivo*. In a mouse model, we also demonstrate that the homogeneous anti-EGFR mAb–Ang2 conjugate administered intravenously efficiently accumulates in intracranial tumors. These findings suggest that homogeneous conjugation of BBB shuttle peptides such as Ang2 is a promising approach to enhancing the therapeutic efficacy of antibody agents for CNS diseases.

## Abstract 2

Homogeneous conjugation of angiopep-2 to a monoclonal antibody improves binding affinity for brain microvascular endothelial cells and accumulation into brain tissues and tumors across the BBB.

## Graphical abstract

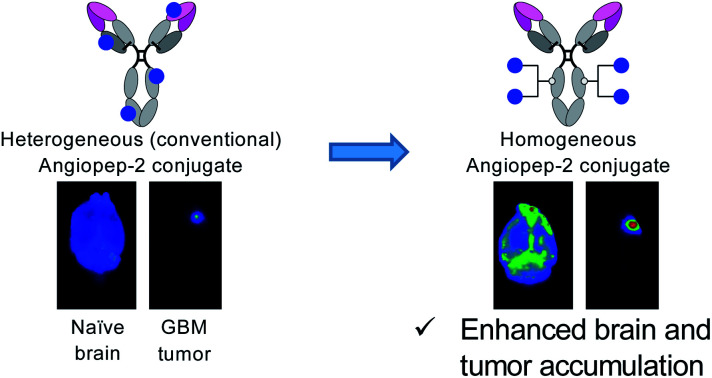

## Introduction

Antibody-based therapy has shown great success in the treatment of many diseases, including cancers.^1^ The use of mAbs has also been extensively evaluated in treating disorders of the central nervous system (CNS).^2^ A recent successful example in this field is the amyloid beta-targeting monoclonal antibody (mAb) aducanumab (Aduhelm^®^) approved for treating Alzheimer's disease by the Food and Drug Administration (FDA) in 2021.^3^ Nevertheless, many studies have led to a consensus that the therapeutic efficacy of most mAbs for CNS diseases is substantially limited due to their extremely low permeability to the brain. Brain tissues are protected by the blood–brain barrier (BBB), which consists of microvascular endothelial cells connected by the tight junctions, pericytes, and astrocytes.^4^ While the BBB plays a role in maintaining brain homeostasis by blocking potentially harmful endogenous and exogenous molecules, this cellular border tightly restricts drug permeability into the brain. Contrary to a common belief, a recent study has demonstrated that an intact BBB also exists in glioblastoma multiforme (GBM) tumors and can block an influx of mAbs and antibody–drug conjugates (ADCs).^5^ Thus, improvement in drug delivery efficiency and BBB permeability of therapeutic antibodies and ADCs is critically needed to advance this drug class further for the effective treatment of CNS diseases.

A plethora of molecular design and administration methods have been investigated to improve mAb delivery to the brain parenchyma.^6^ Modification with BBB shuttle peptides is a common approach for promoting transcytosis mediated by transporters or receptors on brain endothelial cells.^7^ Angiopep-2 (Ang2), a 19-mer synthetic BBB shuttle peptide,^8^ has been shown to promote brain uptake of small molecules,^9,10^ liposomes,^11–13^ and nanoparticles^14–16^*via* transcytosis mediated by the low-density lipoprotein receptor-related protein 1 (LRP-1).^17,18^ Further, Régina *et al.* reported that an anti-HER2 mAb–Ang2 conjugate showed enhanced BBB penetrability and therapeutic efficacy in an intracranial tumor mouse model.^19^ While promising, they used a heterogeneous conjugate that differs in conjugation sites and the number of Ang2 installed. We hypothesized that the transcytosis efficiency of Ang2 conjugates could be further improved by modifying the conjugation modality. Herein, we report that a homogeneous mAb–Ang2 conjugate shows higher binding affinity for LRP-1 and enhanced accumulation into normal brain tissues in healthy mice compared with a heterogeneous variant. We also demonstrate that the homogeneous Ang2 conjugate administered intravenously efficiently targets intracranially implanted GBM tumors. Our findings could lay the foundation for developing better antibody-based therapeutics for CNS disorders.

## Results and discussion

### Construction of a homogeneous mAb–Ang2 conjugate

**Fig. 1:**
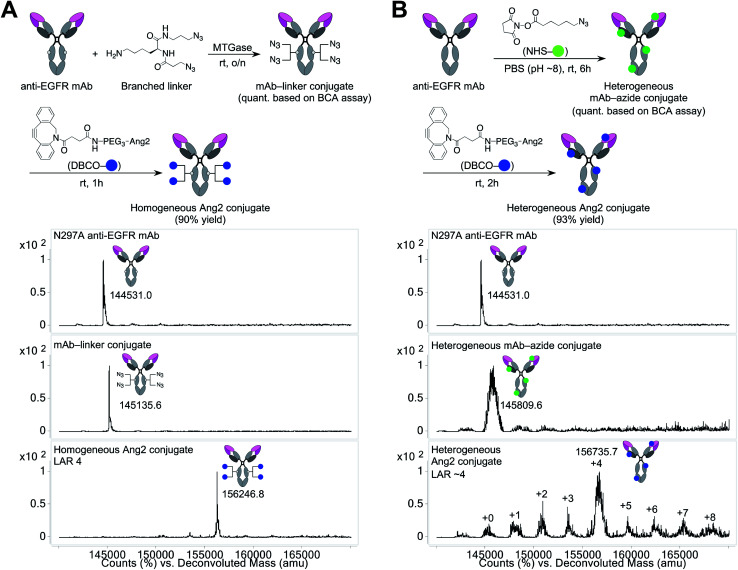
Construction and characterization of mAb–Ang2 conjugates. (A) Preparation and ESI-MS analysis of homogeneous mAb–Ang2 conjugate. Top panel: N297A anti-EGFR mAb. Middle panel: mAb–linker conjugate. Bottom panel: homogeneous mAb–Ang2 conjugate with a LAR of 4. (B) Preparation and ESI-MS analysis of heterogeneous mAb–Ang2 conjugate. The average LAR was determined to be ∼4 based on the ion intensity of each LAR species. DBCO, dibenzocyclooctyne; Ang2, angiopep-2; LAR, ligand-to-antibody ratio; MTGase, microbial transglutaminase; NHS, *N*-hydroxysuccinimide; PEG, polyethylene glycol.

Using the linker and conjugation technologies we have developed previously,^20–22^ we constructed a homogeneous Ang2–mAb conjugate targeting the epidermal growth factor receptor (EGFR) and its truncated mutant EGFRvIII (Fig. 1A). First, we used microbial transglutaminase (MTGase) to site-specifically install a branched diazide spacer onto the side chain of glutamine 295 (Q295) within the Fc region of the N297A mutated mAb. In general, mAbs have many glutamine residues within their sequence. However, as demonstrated by Jeger *et al.*,^23^ MTGase can specifically modify the side chain of Q295 within aglycosylated human IgG1 due to increased accessibility and flexibility. We have confirmed that this conjugation method can be used for branched spacer installation.^20–22^ Using this conjugation method, we could generate homogeneous conjugates containing diazide spacers at Q295 in a quantitative manner.

To install Ang2 peptides to the linker conjugate, we synthesized a dibenzocyclooctyne (DBCO)-functionalized Ang2 module (DBCO–Ang2) by solid-phase peptide synthesis followed by liquid-phase DBCO installation on the N-terminus using a DBCO-NHS ester (see ESI†). Most Ang2-functionalized molecules have been prepared by cysteine–maleimide alkylation between Ang2 and a delivery vehicle. Although this method is simple, cysteine–maleimide adducts have been shown to undergo deconjugation through thiol exchange with serum proteins, leading to a partial loss of conjugated molecules in circulation.^24^ This problem can be solved by amide coupling using NHS esters; however, Ang2 contains two lysine residues within the peptide sequence. If these amines are used to introduce DBCO, capped lysine residues may reduce Ang2–LRP1 interactions due to the loss of cationic chains. Thus, to install DBCO on the N-terminus without lysine capping, the primary amines on the two lysine residues need to be differentiated transiently from that on the N-terminus. However, the DBCO group degrades in the presence of strong acids such as 95% TFA, which is generally used to cleave peptides from Rink amide resin in Fmoc solid-phase peptide synthesis. To overcome these issues, we established a new synthetic route. First, we replaced the Fmoc-protecting group on the N-terminus of Ang2 with a Boc group on resin. Subsequently, the Alloc-protecting groups on the two lysine residues were replaced with Fmoc. After cleaving the peptide from the resin with 95% TFA (10% overall yield for the entire solid-phase synthesis), we installed DBCO on the N-terminus using a DBCO-NHS ester in the liquid phase. Finally, we performed Fmoc deprotection under basic conditions. Diethylamine is most commonly used for liquid-phase Fmoc deprotection; however, in our case, a diethylamine–Fmoc adduct was formed and found to be inseparable in reverse-phase HPLC purification. After screening several conditions, we discovered that an aqueous NaOH solution efficiently afforded the desired product (DBCO–Ang2) without complication by byproducts (71% yield for the DBCO installation and Fmoc removal steps).

The DBCO–Ang2 module was mixed with the linker conjugate to yield a homogeneous Ang2–mAb conjugate with a ligand-to-antibody ratio (LAR) of 4 (90% yield). We also prepared a heterogeneous Ang2–mAb conjugate from the same parent N297A mAb by conventional lysine coupling;^19^ after 6-azido-hexanoic acid *N*-hydroxysuccinimide (NHS) ester was stochastically installed to solvent-accessible lysine residues, the DBCO–Ang2 module was added to give the heterogeneous variant (Fig. 1B). ESI-MS analysis showed that the average LAR of this conjugate was 4, with a distribution ranging from 0 to 8. Size-exclusion chromatography analysis showed that both homogeneous and heterogeneous conjugates existed predominantly in the monomeric form (>99%, Fig. S1†).

### Evaluation of *in vitro* binding affinity and cytotoxicity

**Fig. 2:**
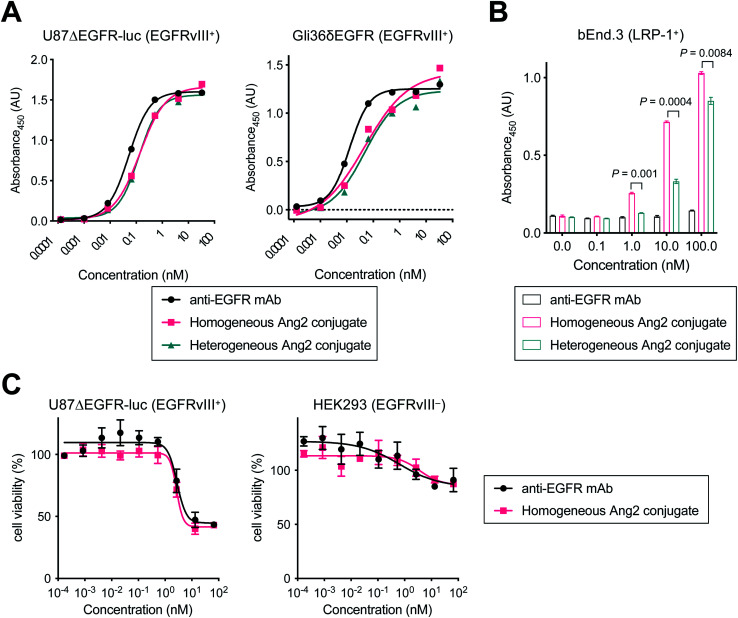
Evaluation of binding affinity and cytotoxicity *in vitro*. Cell-based ELISA in (A) U87ΔEGFR-Luc (EGFRvIII^+^), Gli36δEGFR (EGFRvIII^+^) and (B) bEnd.3 (LRP-1^+^) cells. (C) Cell viability assay in U87ΔEGFR-Luc (EGFRvIII^+^) and HEK293 (EGFRvIII^−^) cells. Concentrations are based on the antibody dose without normalizing to each LAR. All assays were performed in triplicate. Data are presented as mean values ± SEM. Statistical analysis was performed using Welch's *t*-test (two-tailed, unpaired, uneven variance).

Next, we tested the Ang2 conjugates for binding affinity for EGFRvIII and LRP-1 by cell-based enzyme-linked immunosorbent assay (ELISA). The GBM cell lines U87ΔEGFR-Luc and Gli36δEGFR cells were used for EGFRvIII binding, and the murine brain endothelial cell line bEnd.3 was used for LRP-1 binding (Fig. 2 and Table 1). Both homogeneous and heterogeneous Ang2 conjugates showed approximately >2-fold higher *K*_D_ values in U87ΔEGFR-Luc (0.115 and 0.122 nM, respectively) than that of the unmodified anti-EGFR mAb (0.053 nM). The same trend was also observed in Gli36δEGFR cells (*K*_D_: 0.024 nM, homogeneous conjugate, 0.030 nM, heterogeneous conjugate, 0.012 nM, unmodified mAb). Although site-specifically installed onto the Fc region distal from the Fab moiety, these results indicate that the 19-mer Ang2 peptide partially impaired the mAb–receptor interactions due to its large molecular size. While the parent anti-EGFR mAb did not bind to LRP-1-positive bEnd.3 cells, both Ang2 conjugates strongly bound to the cells (Fig. 2B). *K*_D_ values were 4.96 nM (homogeneous Ang2 conjugate) and 28.5 nM (heterogeneous variant), respectively (Table 1). This result demonstrates that our molecular design provides the anti-EGFR mAb with dual-targeting functionality for EGFRvIII and LRP-1. Furthermore, compared to conventional heterogeneous conjugation, homogeneous conjugation of Ang2 peptides provides improved binding affinity for LRP-1 without significantly impairing the binding affinity and specificity of the parent mAb for EGFRvIII. These findings suggest that homogeneous Ang2 conjugation can potentially help promote LRP-1 receptor-mediated transcytosis more effectively.

**Table 1:** *K*_D_ values of unmodified N297A anti-EGFR mAb and Ang2 conjugates in human GBM cell lines and a murine brain endothelial cell line (*n* = 3). Values in parentheses are 95% confidential intervals

	*K*_D_ (nM)		
	anti-EGFR mAb	Homogeneous Ang2 conjugate	Heterogeneous Ang2 conjugate
U87ΔEGFR-luc	0.053 (0.048–0.059)	0.115 (0.104–0.127)	0.122 (0.101–0.148)
Gli36δEGFR	0.012 (0.010–0.015)	0.024 (0.021–0.029)	0.030 (0.026–0.035)
bEnd.3	Not determined	4.96 (4.52–5.44)	28.5 (22.7–36.8)

To investigate whether conjugating Ang2 peptides onto the mAb affects cytotoxicity in antigen-positive GBM cells and antigen-negative normal cells, we performed cytotoxicity assays for unmodified anti-EGFR mAb and the homogeneous Ang2 conjugate in both U87ΔEGFR-luc and HEK293 cells (Fig. 2C). These conjugates exhibited similar dose-dependent responses in both antigen-positive and -negative cells. These results demonstrate that Ang2 conjugation does not alter the cytotoxicity or target specificity profiles of the parent mAb.

### Biodistribution of Ang2–mAb conjugates in healthy mice and intracranial tumor-bearing mice

We evaluated using mouse models whether homogeneous Ang2 conjugation to mAbs improves accumulation into brain tissues across the BBB. To visualize tissue biodistribution, we fluorescently labeled the parent anti-EGFR mAb, the homogeneous Ang2 conjugate, and the heterogeneous variant using Cy5.5-NHS ester. To investigate the multiplicity effect, a highly loaded homogeneous Ang2–mAb conjugate (LAR of 8) was also constructed using N297Q-mutated anti-EGFR mAb (Fig. S2†). Healthy CD-1 mice were then injected intravenously with each Cy5.5 conjugate at 3 mg kg^−1^. Subsequently, conjugates circulating or bound on brain microvascular endothelial cells were removed by cardiac perfusion with PBS. As a large part of antibodies administered remains circulating in 24 h post-injection, cardiac perfusion is commonly performed right before harvesting brains to avoid false-positive readout.^14,15,19^

**Fig. 3:**
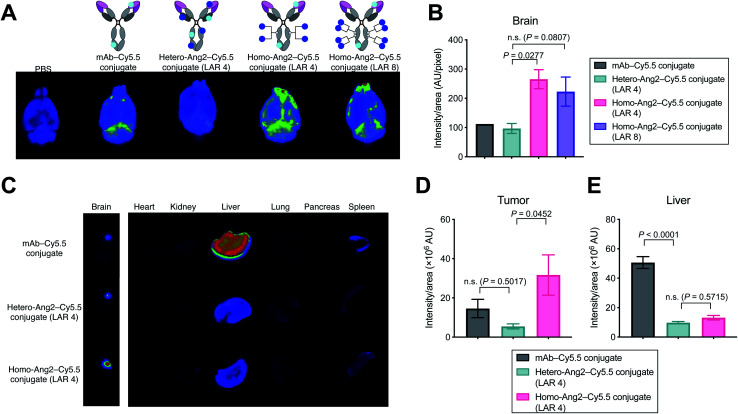
Biodistribution studies in healthy mice and tumor-bearing mice. (A) *Ex vivo* fluorescence images of whole brains harvested from CD-1 mice 2 hours after intravenous injection of each fluorescent conjugate at 3 mg kg^−1^ (*n* = 1 for PBS and mAb–Cy5.5 conjugate; *n* = 3 for other groups). (B) Semi-quantification of the Cy5.5 signal in the whole brains. mAb–Cy5.5 conjugate (black), heterogeneous mAb–Ang2–Cy5.5 conjugate (LAR 4, green), homogeneous mAb–Ang2–Cy5.5 conjugate (LAR 4, magenta), homogeneous mAb–Ang2–Cy5.5 conjugate (LAR 8, purple). (C) Biodistribution in orthotopic Gli36δEGFR tumor-bearing mice (3 mg kg^−1^, athymic nude, *n* = 4 for mAb–Cy5.5 conjugate; *n* = 3 for other groups) at 24 h after intravenous injection. *Note: color contrast of the brain panel is enhanced for clarity*. (D and E) Semi-quantification of the Cy5.5 signal derived from the tumor lesions in the whole brains (D) and from the liver (E). mAb–Cy5.5 conjugate (black), heterogeneous mAb–Ang2–Cy5.5 conjugate (LAR 4, green), homogeneous mAb–Ang2–Cy5.5 conjugate (LAR 4, magenta). A representative image from each group is shown in all panels of fluorescence images. Signal intensity was calculated using Image J software. Data are presented as mean values ± SEM. For statistical analysis, a one-way ANOVA with a Dunnett's post hoc test (control: heterogeneous mAb–Ang2–Cy5.5 conjugate) was used.

We have shown that the brain accumulation of antibody conjugates reaches a very high to the maximal level 24 h post injection.^25^ Based on this finding, we first performed initial testing and *ex vivo* quantification 24 h post-injection. However, we did not observe a significant difference (*P* value of <0.05 or >2-fold in intensity) in brain accumulation in naïve mice between unmodified mAb and our homogeneous Ang2 conjugate (Fig. S3†). We then performed the same study with *ex vivo* imaging 2 h post injection. *Ex vivo* fluorescence imaging of brain tissues revealed that the homogeneous Ang2 conjugate with a LAR of 4 accumulated in the brain parenchyma more effectively than the heterogeneous variant (*P* = 0.0277, Fig. 3A and B). Unexpectedly, increasing LAR from 4 to 8 did not improve but instead slightly reduced the accumulation in the brain. Although in-depth studies are needed for clarification, this result suggests that over-conjugation of Ang2 can cause detrimental effects on other parameters such as ligand–receptor interactions, circulation stability, and/or clearance rate.

Finally, we performed a biodistribution study using a brain tumor-bearing mouse model. Mice bearing intracranial Gli36δEGFR tumors were administered intravenously with each dye conjugate at 3 mg kg^−1^. After 24 h, major organs including brains were harvested for imaging. Gratifyingly, the homogeneous Ang2 conjugate showed significantly increased tumor accumulation compared to the heterogeneous variant (*P* = 0.0452). The heterogeneous conjugate did not show a statistically significant increase in brain tissue retention compared to the parent mAb (*P* = 0.5017, Fig. 3C and D). Although Régina *et al.* reported that an anti-HER2 mAb–heterogeneous Ang2 conjugate administered intravenously at 10 mg kg^−1^ showed effective intracranial tumor accumulation,^19^ we did not observe such effect for our heterogenous Ang2 conjugate at 3 mg kg^−1^. Of particular note, liver accumulation of both Ang2 conjugates was markedly attenuated (Fig. 3C and E). In general, most antibodies and antibody–drug conjugates administered are taken up by the liver, releasing highly active catabolites.^26^ Therefore, decreased liver accumulation of antibody conjugates could help limit undesired systemic toxicity. Our findings demonstrate that homogeneous conjugation of Ang2 is advantageous over conventional heterogeneous conjugation for enhancing BBB permeability and brain tumor targeting while minimizing potential toxicity.

Most l-amino acid-based peptides with flexible structures are unstable and degrade in circulation over time (*T*_1/2_: ∼30 min). Although the conjugation to an mAb may confer some protective effect, the conjugated Ang2 likely has a short half-life *in vivo*. As such, loss of the Ang2 may be a reason for the limited improvement in BBB penetration observed in the naïve mouse model. On the other hand, in GBM tumor-bearing mice, we could observe enhanced accumulation in the brain tumor for our homogeneous Ang2 conjugate even at 24 h post injection. We speculate that binding of the mAb moiety to its target receptor on the surface of GBM cells (EGFRvIII in our case) allowed for extended retention of the conjugate in the brain tumor upon BBB penetration. Along with our cysteine–maleimide linkage-free design, structural modifications to Ang2 for improving stability may enhance the durability of its BBB permeation effect in healthy brain tissues. Nonetheless, a solid conclusion drawn from these studies is that homogeneous Ang2 conjugation is a better approach to improving BBB penetration than conventional heterogeneous conjugation.

## Conclusions

This study demonstrates that homogeneous conjugation of Ang2 enhances the binding affinity for LRP-1-positive cells *in vitro* and improves accumulation into the brain tissues and intracranial tumors *in vivo*. These findings could also be true for other BBB shuttle peptides targeting other transporters or receptors (*e.g.*, transferrin receptor). We believe that further studies of physicochemical properties, *in vivo* stability, therapeutic efficacy, and safety profiles will provide optimal molecular design, which could eventually lead to promising antibody conjugates with high therapeutic potential for treating CNS diseases. Based on our recent findings that homogeneous antibody–drug conjugation improves delivery of conjugated drugs and therapeutic efficacy for intracranial tumors,^25^ such efforts may also pave a path to effective therapy for brain tumors, including GBM.

## Author contributions

Conceptualization, K. T.; methodology, Y. A., W. X., N. Z., Z. A., and K. T.; validation, Y. A., and W. X.; formal analysis, Y. A.; investigation, Y. A., W. X., A. Y., C. M. Y., and K. T.; resources, N. Z., Z. A., and K. T.; writing – original draft, Y. A., and K. T.; writing – review & editing, Y. A., and K. T.; visualization, Y. A.; supervision, K. T.; project administration, K. T.; funding acquisition, Y. A., A. Y., Z. A., and K. T.

## Conflicts of interest

Y. A., C. M. Y., N. Z., Z. A., and K. T. are named inventors on a patent application relating to the work filed by the Board of Regents of the University of Texas System (PCT/US2018/034363; US-2020-0115326-A1; EU18804968.8-1109/3630189). The remaining authors declare no competing interests.

## Supplementary Material

RA-012-D1RA08131D-s001
