# Supplementary material for: Homogeneous antibody–angiopep 2 conjugates for effective brain targeting
Source: RSC Adv. 2022 Jan 26;12(6):3359–64. doi: 10.1039/d1ra08131d (PMC8979263; doi:10.1039/d1ra08131d)
Supplement: RA-012-D1RA08131D-s001 [file RA-012-D1RA08131D-s001.pdf]

Electronic Supplementary Material (ESI) for RSC Advances.
This journal is © The Royal Society of Chemistry 2022

Supplementary Information

Homogeneous antibody-angiopep 2 conjugates for effective brain targeting

Yasuaki Anami, Wei Xiong, Aiko Yamaguchi, Chisato M Yamazaki, Ningyan Zhang, Zhiqiang An, and Kyoji Tsuchikama*

Texas Therapeutics Institute, The Brown Foundation Institute of Molecular Medicine, McGovern Medical School, The University of Texas Health Science Center at Houston, Houston, TX 77054, USA.

*To whom correspondence should be addressed: Kyoji Tsuchikama (Kyoji.Tsuchikama@uth.tmc.edu)

Supplementary Materials and Methods

General information

Unless otherwise noted, all materials for chemical synthesis were purchased from commercial suppliers (Acros Organics, AnaSpec, Broadpharm, Chem-Impex International, Fisher Scientific, Levena Biopharma, Sigma Aldrich, TCI America, and other vendors) and used as received. All anhydrous solvents were purchased and stored over activated molecular sieves under argon atmosphere.

Analytical reverse-phase high performance liquid chromatography (RP-HPLC) was performed using an Agilent LC-MS system consisting of a 1100 HPLC and a 1946D single quadrupole electrospray ionization (ESI) mass spectrometer equipped with a C18 reverse-phase column (Accucore™ C18 column, 3 × 50 mm, 2.6 µm, Thermo Scientific) or a Thermo LC-MS system consisting of a Vanquish UHPLC and a LTQ XL™ linear ion trap mass spectrometer equipped with a C18 reverse-phase column (Accucore™ Vanquish™ C18+ UHPLC column, 2.1 × 50 mm, 1.5 µm, Thermo Scientific). Standard analysis conditions for organic molecules were as follows: flow rate = 0.5 mL/min (for both systems); solvent A = water containing 0.1% formic acid; solvent B = acetonitrile containing 0.1% formic acid. Compounds were analyzed using a linear gradient and monitored with UV detection at 210 and 254 nm. Preparative HPLC was performed using a Breeze HPLC system (Waters) equipped with a C18 reverse-phase column (XBridge Peptide BEH C18 OBD Prep Column, 130Å, 5 µm, 19 × 150 mm, Waters). Standard purification conditions were as follows: flow rate = 20 mL/min; solvent A = water containing 0.05% trifluoroacetic acid (TFA), 0.1% formic acid or 0.1% NH_4OH; solvent B = acetonitrile containing 0.05% TFA (standard conditions), 0.1% formic acid (FA conditions), or 0.1% NH_4OH (basic conditions). Compounds were analyzed using a linear gradient and monitored with UV detection at 210 and 254 nm. In all cases, fractions were analyzed off-line using either of the LC-MS systems for purity confirmation and those containing a desired product were lyophilized using a Labconco Freezone 4.5 Liter Benchtop Freeze Dry System. High-resolution mass spectra were obtained using a Thermo Q Exactive™ Hybrid Quadrupole-Orbitrap™ Mass Spectrometer.

Synthesis

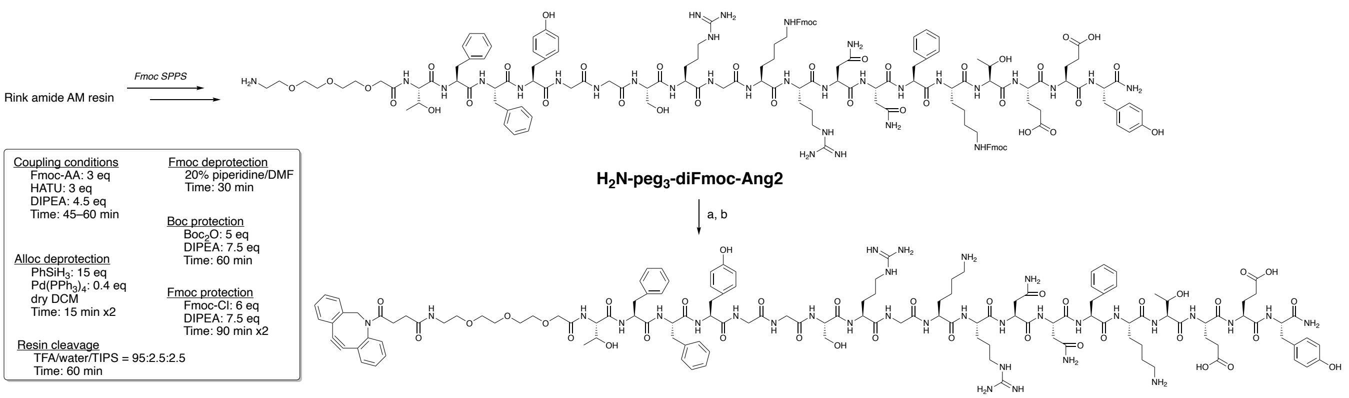

DBCO-Ang2

Synthesis of DBCO–Ang2 module. Reagents and conditions: (a) DBCO-NHS, DIPEA, DMF, rt, 3 h. (b) NaOH, rt, 1h.

Preparation of H_2N-peg$_3$-diFmoc-Ang2 by Fmoc Solid-Phase Peptide Synthesis (Fmoc SPPS)

Rink amide AM resin was taken to a manual solid-phase reactor and treated with 20% piperidine/DMF (5 mL) for 20 min to remove an Fmoc-protecting group. Then the solution was drained and the resin was washed with DMF (5×3 mL) and DCM (5×3 mL). Fmoc-protected amino acid (3 equiv.) was pre-activated using 1-[bis(dimethylamino)methylene]-1H-1,2,3-triazolo[4,5-b]pyridinium 3-oxid hexafluorophosphate (HATU, 3 equiv.) and diisopropylethylamine (DIPEA, 4.5 equiv.) in DMF for 3 min, and the cocktail was added to the resin. The resin was agitated at room temperature for 45–60 min. The completion of the coupling was verified by the Kaiser test. After each coupling step, the coupling cocktail was drained and the resin was washed with DMF (5×3 mL) and DCM (5×3 mL). To remove the Fmoc-protecting group after each coupling, the resin was treated with 20% piperidine/DMF (5 mL). After elongation of the peptide, the Fmoc-protecting group on the *N*-terminus was replaced with a Boc-protecting group and Alloc-protecting groups on the side chain of Lysine residues were swapped to Fmoc. To protect *N*-terminal amine with the Boc-protecting group, the resin was treated with Boc_2O (5 equiv.) and DIPEA (7.5 equiv.) in DMF (5 mL) and agitated at room temperature for 60 min. To remove the Alloc-protecting group, the resin was treated with $Pd(PPh_3)_4$ (0.4 equiv.) and phenylsilane (15 equiv.) in DCM (5 mL) under Ar atmosphere (15 min×2) and washed with DMF (5×3 mL) and DCM (5×3 mL). For Fmoc protection, the resin was treated with Fmoc-Cl (6 equiv.) and DIPEA (7.5 equiv.) in DMF (5 mL) and agitated at room temperature for 90 min. This step was repeated twice. The resulting protected peptide resin was treated with cocktail of trifluoroacetic acid (TFA)/water/triisopropyl silane (TIPS) (95:2.5:2.5) at room temperature for 1 h. The solution was concentrated in vacuo and the crude peptide was precipitated with cold diethyl ether (20 mL) followed by centrifugation at 2,000 ×g for 3 min (3 times). The resulting crude peptide was purified by preparative RP-HPLC under standard conditions to afford analytically pure

peptide **H_2N-peg$_3$-diFmoc-Ang2** (33 mg, 10%). Purity was confirmed by LC-MS. White powder. HRMS (ESI) Calcd. For $C_{142}H_{188}N_{31}O_{38}$ $[M+3H]^{3+}$: 978.4572, Found: 978.4590.

Preparation of DBCO–Ang2

DBCO-NHS (3.3 mg, 8.2 μmol) and DIPEA (2.2 μL, 12.5 μmol) were added to a solution of **H_2N-peg$_3$-diFmoc-Ang2** (18.4 mg, 6.3 μmol) in DMF (1.5 mL) and the mixture was stirred at room temperature for 3 h. Then, 1*N*-NaOH aq. was added to the reaction mixture and the mixture was stirred at room temperature for another 1 h. The crude products were purified by preparative RP-HPLC under FA conditions to afford analytically pure peptide **DBCO–Ang2** (12.3 mg, 71% for the 2 steps). Purity was confirmed by LC-MS. White powder. HRMS (ESI) Calcd. For $C_{131}H_{181}N_{32}O_{36}$ $[M+3H]^{3+}$: 926.1100, Found: 926.1080.

MTGased-mediated antibody–linker conjugation

Anti-EGFR mAb with N297A mutation (222 µL in PBS, 9.03 mg/mL, 2.0 mg antibody) was incubated with the diazide branched linker developed by us previously[1–5] (5.9 µL of 100 mM stock in water, 80 equiv.) and Activa TI® (58 µL of 40% solution in PBS, Ajinomoto, purchased from Modernist Pantry) at room temperature for 16 h. The reaction was monitored using an Agilent LC-MS system consisting of a 1100 HPLC and a 1946D single quadrupole ESI mass spectrometer equipped with a MabPac RP column (3 × 50 mm, 4 µm, Thermo Scientific). Elution conditions were as follows: mobile phase A = water (0.1% formic acid); mobile phase B = acetonitrile (0.1% formic acid); gradient over 6.8 min from A:B = 75:25 to 1:99; flow rate = 0.4 mL/min. The conjugated antibody was purified by SEC (Superdex 200 increase 10/300 GL, GE Healthcare, solvent: PBS, flow rate = 0.6 mL/min) to afford an antibody–linker conjugate [2.1 mg, quantitative yield determined by bicinchoninic acid (BCA) assay].

Construction of homogeneous antibody–Ang2 conjugate

DBCO–Ang2 (6.4 µL of 10 mM stock solution in DMSO, 1.5 equivalent per azide group) was added to a solution of the mAb–linker conjugate in PBS (300 µL, 5.33 mg/mL), and the mixture was incubated at room temperature for 1 h. The reaction was monitored using Agilent LC-MS system equipped with a MabPac RP column (see above) and the crude products were purified by SEC to afford homogeneous mAb–Ang2 conjugate with LAR of 4 (1.44 mg, 90% yield determined by BCA assay). Analysis and purification conditions were the same as described above. Homogeneity was confirmed by ESI-MS analysis. Homogeneous mAb–Ang2 conjugate with LAR of 8 was prepared in the same manner using anti-EGFR mAb with N297Q mutation.

Construction of heterogeneous antibody–Ang2 conjugate

Aglycosylated anti-EGFR mAb (833 µL in PBS, 3.0 mg/mL, 2.5 mg antibody) was mixed with 1 M phosphate solution at pH 9 (83.3 µL) and 6-azidohexanoic acid NHS ester (40 µL of 5 mM stock solution in DMSO, 12 equiv.) and the mixture was incubated at room temperature for 6 h. The reaction was monitored using Agilent LC-MS system equipped with a MabPac RP column (see above). The crude products were purified by SEC to afford heterogeneous mAb–azide conjugate (2.7 mg, quantitative yield determined by BCA assay). Then DBCO–Ang2 (7.1 µL of 10 mM stock solution in DMSO, 4.5 equiv.) was added to a solution of the heterogeneous mAb–azide conjugate in PBS (350 µL, 6.78 mg/mL), and the mixture was incubated at room temperature for 2 h. The reaction was monitored using Agilent LC-MS system equipped with a MabPac RP column (see above) and the crude products were purified by SEC to afford heterogeneous mAb–Ang2 conjugate (2.2 mg, 93% yield determined by BCA assay). Analysis and

purification conditions were the same as described above. The average LAR value was estimated based on ion intensity of each LAR species in ESI-MS analysis.

Cell lines

U87ΔEGFR-luc was generated by lentiviral transduction of U87ΔEGFR cells (a gift from Dr. Balveen Kaur, UTHealth) using Lentifect™ lentiviral particles encoding for firefly luciferase and a puromycin-resistant gene (GeneCopoeia, LP461-025). Transduction was performed according to the manufacturer's instruction. Gli36δEGFR (a gift from Dr. Balveen Kaur, UTHealth), U87ΔEGFR-luc, HEK293 (ATCC), and bEnd.3 (ATCC) were cultured in DMEM (Corning) supplemented with 10% EquaFETAL®(Atlas Biologicals), GlutaMAX® (2 mM, Gibco), and penicillin-streptomycin (penicillin: 100 units/mL; streptomycin: 100 μg/mL, Corning). All cells were cultured at 37 °C under 5% CO_2 and passaged before becoming fully confluent up to 30 passages. All cell lines were periodically tested for mycoplasma contamination.

Cell-based ELISA

Cells (U87ΔEGFR-luc, Gli36δEGFR or bEnd.3) were seeded in a culture-treated 96-well clear plate (10,000 cells/well in 100 μL culture medium) and incubated at 37 °C with 5% CO2 for 24 h. Paraformaldehyde (8%, 100 μL) was added to each well and incubated for 15 min at room temperature. The medium was discarded and the cells were washed three times with 100 μL of PBS. Cells were treated with 100 μL of blocking buffer (0.2% BSA in PBS) with agitation at room temperature for 2 h. After the blocking buffer was discarded, serially diluted samples (in 100 μL PBS containing 0.1% BSA) were added and the plate was incubated overnight at 4 °C with agitation. The buffer was discarded and the cells were washed three times with 100 μL of PBS containing 0.25% Tween 20. Cells were then incubated with 100 μL of donkey anti-human IgG–HRP conjugate (diluted 1:10,000 in PBS containing 0.1% BSA) was added and the plate was incubated at room temperature for 1 h. The plate was washed three times with PBS containing 0.25% Tween 20, and 100 μL of 3,3′,5,5′-tetramethylbenzidine (TMB) substrate (0.1 mg/mL) in phosphate–citrate buffer/30% H_2O_2 (1:0.0003 volume to volume, pH 5) was added. After color was developed for 10−30 min, 25 μL of 3 N-HCl was added to each well and then the absorbance at 450 nm was recorded using a plate reader (BioTek Synergy HTX). K_D values were then calculated using Graph Pad Prism 8 software. All assays were performed in triplicate. Statistical analysis was performed using Welch's *t*-test (two-tailed, unpaired, uneven variance).

Cell viability assay

Cells were seeded in a culture-treated 96-well clear plate (5,000 cells/well in 50 μL culture medium) and incubated at 37 °C under 5% CO2 for 24 h. Serially diluted samples (50 μL) were added to each well and the plate was incubated at 37 °C for 72 h. After the old medium was replaced with 100 μL fresh medium, 20 μL of a mixture of WST-8 (1.5 mg/mL, Cayman chemical) and 1-methoxy-5-methylphenazinium methylsulfate (100 μM, Cayman Chemical) was added to each well, and the plate was incubated at 37 °C for 2 h. After gently agitating the plate, the absorbance at 460 nm was recorded using a plate reader (BioTek Synergy HTX). All assays were performed in triplicate.

Animal studies

All procedures were approved by the Animal Welfare Committee of the University of Texas Health Science Center at Houston and performed in accordance with the institutional guidelines for animal care and use. All animals were housed under controlled conditions, namely 21–22 °C (± 0.5 °C), 30–75% (±10%) relative humidity, and 12:12 light/dark cycle with lights on at 7.00 a.m. Food and water were available ad libitum for all animals. NCr nude mice were purchased from Taconic (NCRNU-F sp/sp) and used without in-house breeding. CD-1® mice was purchased from Charles River Laboratories (Strain Code: 022) and used without in-house breeding.

Orthotopic xenograft mouse model of human glioblastoma multiforme

Gli36δEGFR (1×10^5 cells) was stereotactically implanted into NCr nude mice (6–8 weeks old, female) as follows. NCr nude mice were injected intraperitoneally with a cocktail of ketamine (67.5 mg/kg) and dexmedetomidine (0.45 mg/kg) and maintained at 37 °C on a heating pad until the completion of surgery. After the head skin was shaved and treated with 10 μL of 0.25% bupivacaine supplemented with epinephrine (1:200,000), anesthetized mice were placed on a stereotactic instrument. After disinfecting the head skin with chlorhexidine and ethanol, a small incision was made and then a burr hose was drilled into the skull over the right hemisphere (1 mm anterior and 2 mm lateral to the bregma). A 10 μL Hamilton syringe (model 701 N) was loaded with cells suspended in 2 μL cold hanks-balanced salt solution (HBSS) and slowly inserted into the right hemisphere through the burr hole (3.5 mm depth). After a 1-min hold time, cells were injected over a 5-min period (0.4 μL/min). After a 3-min hold time, the needle was retracted at a rate of 0.75 mm/min. The incision was closed using GLUture® (Zoetis) and mice were injected with atipamezole (1 mg/kg, i.p.).

Biodistribution studies

[1] CD-1® mice (5−6 weeks old, male) were administered intravenously with each Cy5.5 conjugate at 3 mg/kg. After 2 h, the mice were anesthetized with ketamine/xylazine. Subsequently, the mice underwent cardiac perfusion with PBS(+) containing sodium heparin (10 units/mL) and 4% paraformaldehyde/PBS(+). Major organs including the brain were then harvested. Cy5.5-based near-infrared fluorescence images of the harvested organs were taken using a LI-COR Odyssey 9120 imager (Ex: 685 nm laser, intensity: 1.0 for brain, L0.5 for other organs, Em: 700 nm channel). Semi-quantification of the signals from ROIs was also performed using LI-COR Image Studio software.

[2] Intracranial Gli36δEGFR tumor-bearing NCr nude mice (6−8 weeks old, female) were prepared as described above. The mice intravenously received each Cy5.5 conjugate at 3 mg/kg 5 days post tumor implantation. After 24 h, the tumor-bearing mice were processed as described above. Major organs including the brain were then harvested. Cy5.5-based near-infrared fluorescence images of the harvested organs were taken using a LI-COR Odyssey 9120 imager (Ex: 685 nm laser, intensity: L0.5 for all organs, Em: 700 nm channel). Semi-quantification of the signals from ROIs was performed using Image J software. Statistical analysis was performed using a one-way ANOVA with Dunnett's post hoc test for multiple comparisons.

LC-MS data

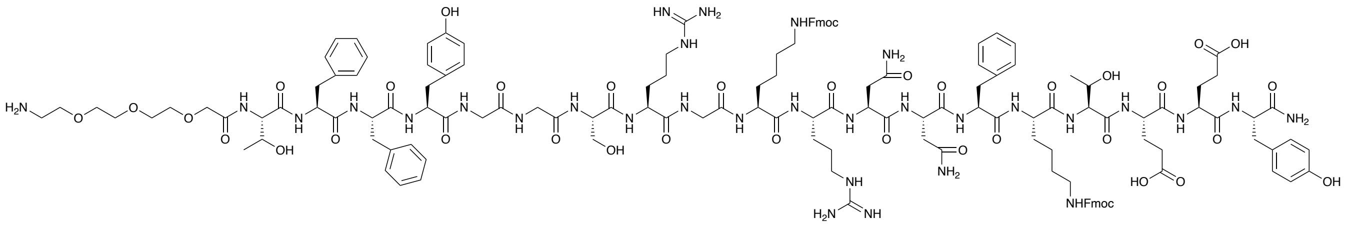

H_2N-peg$_3$-diFmoc-Ang2

Print of all graphic windows

Current Chromatogram(s) Apex Mass Spectrum of Peak 5.327

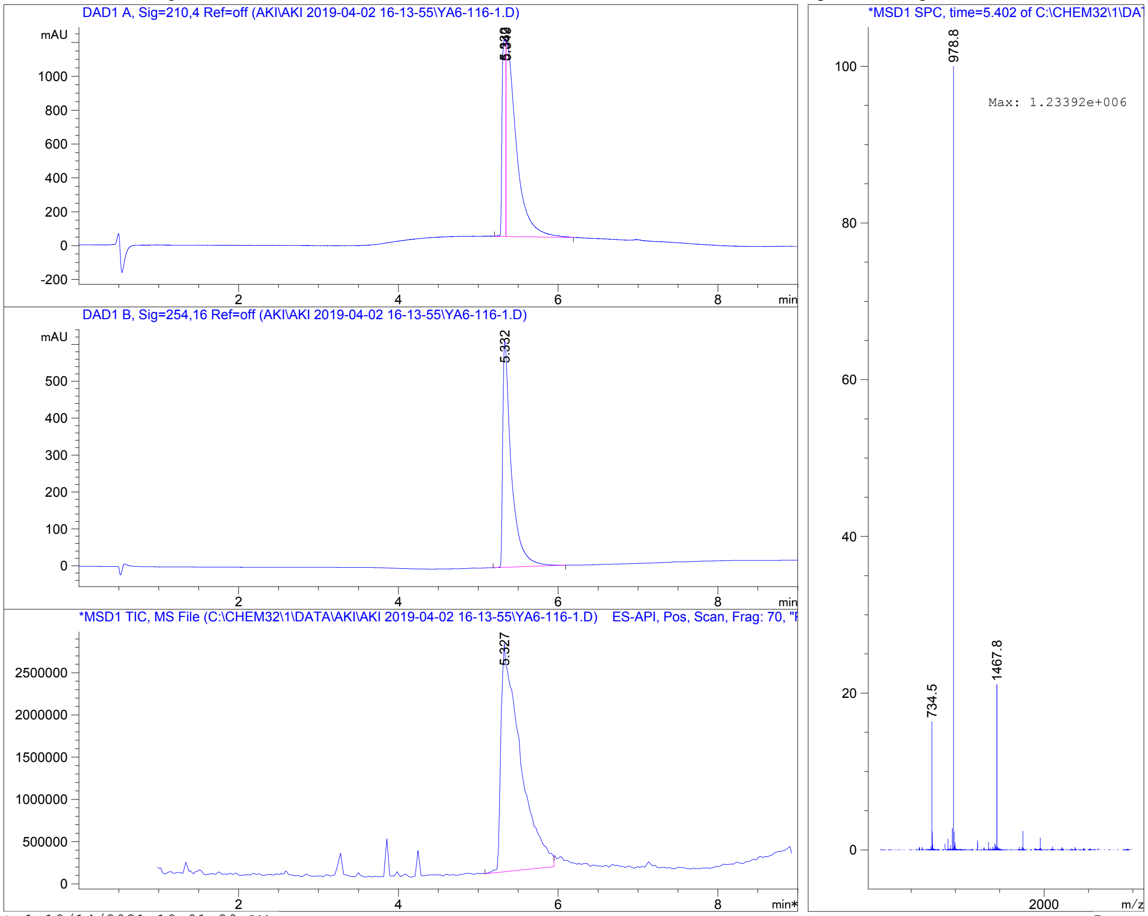

Instrument 1 10/14/2021 10:01:29 AM Page 1 of 1

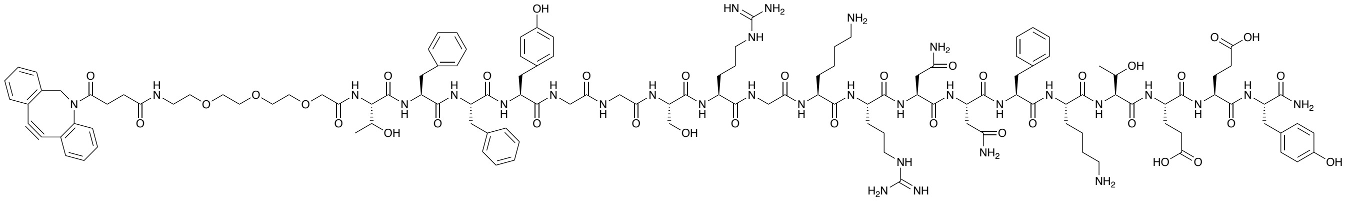

DBCO-Ang2

Print of all graphic windows

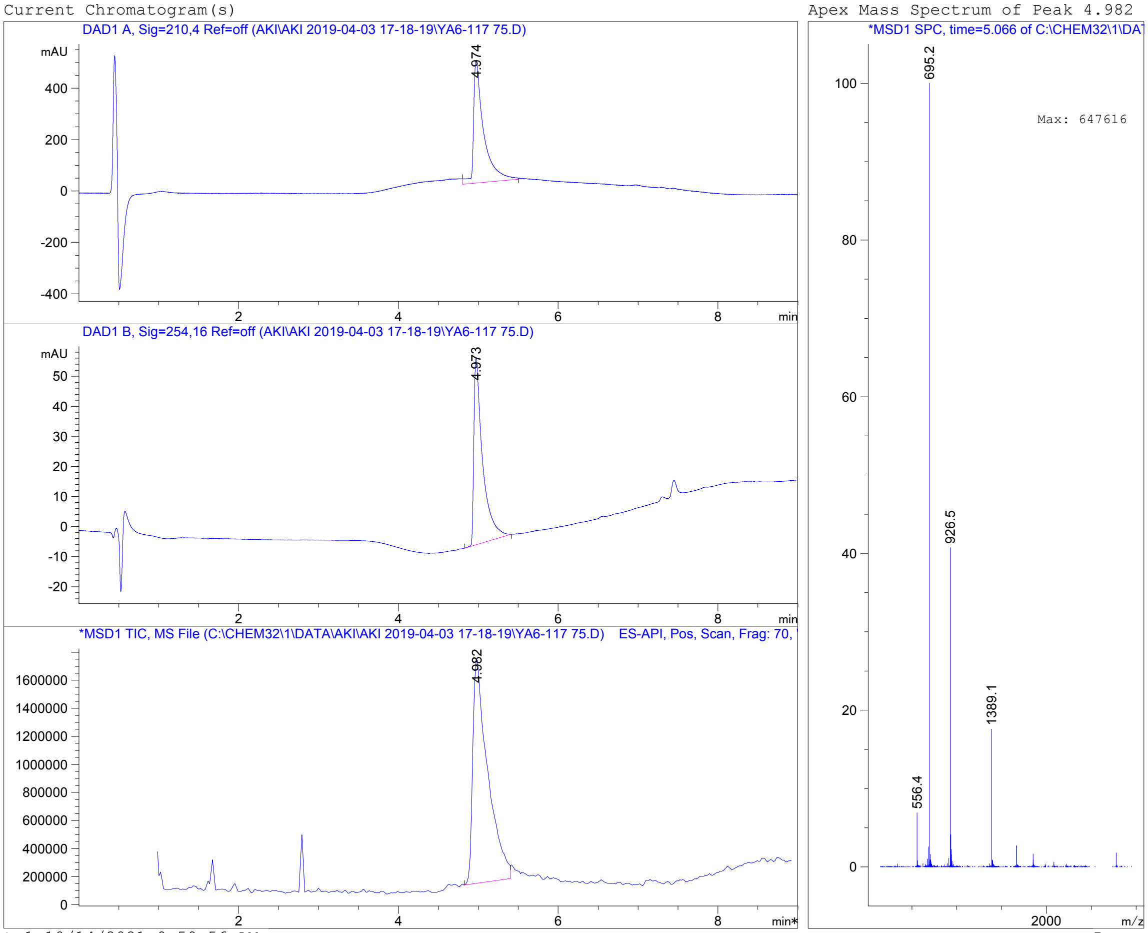

Instrument 1 10/14/2021 9:59:56 AM Page 1 of 1

Supplementary Figures

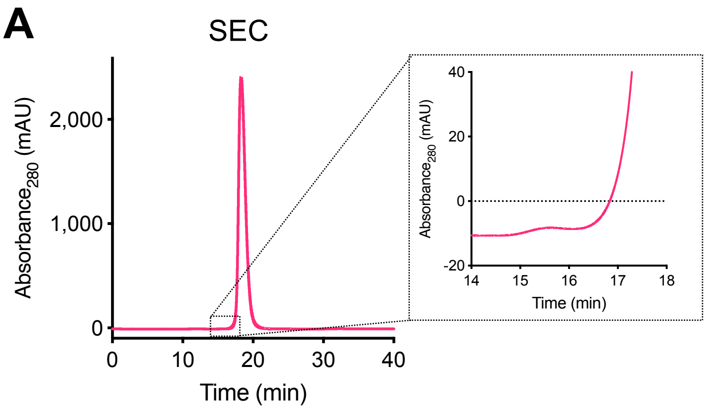

Time (min)	Area%
15.6	0.07
18.3	99.93

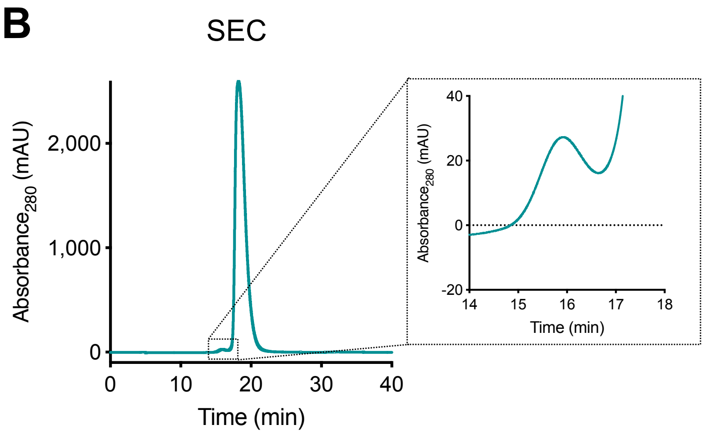

Time (min)	Area%
15.9	0.85
18.2	99.15

Fig. S1. Size-exclusion chromatography (SEC) analysis before purification. SEC traces (UV absorbance: 280 nm) of homogeneous Ang2 conjugate (**A**) and heterogeneous Ang2 conjugate (**B**).

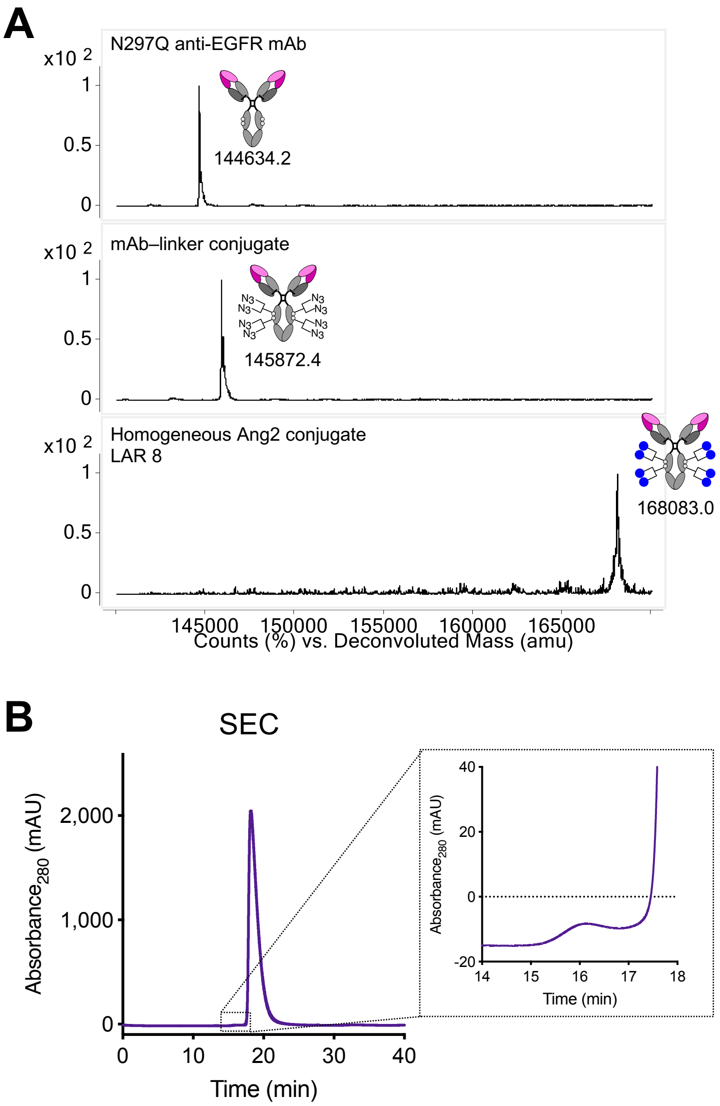

Time (min)	Area%
16.1	0.21
18.2	99.79

Fig. S2. Characterization of homogeneous Ang2 conjugate with LAR of 8. (**A**) Preparation and ESI-MS analysis of homogeneous Ang2 conjugate with LAR of 8. First panel: N297Q anti-EGFR mAb. Second panel: mAb–linker conjugate. Third panel: homogeneous Ang2 conjugate with LAR of 8. (**B**) SEC trace (UV absorbance: 280 nm) of homogeneous Ang2 conjugate with LAR of 8.

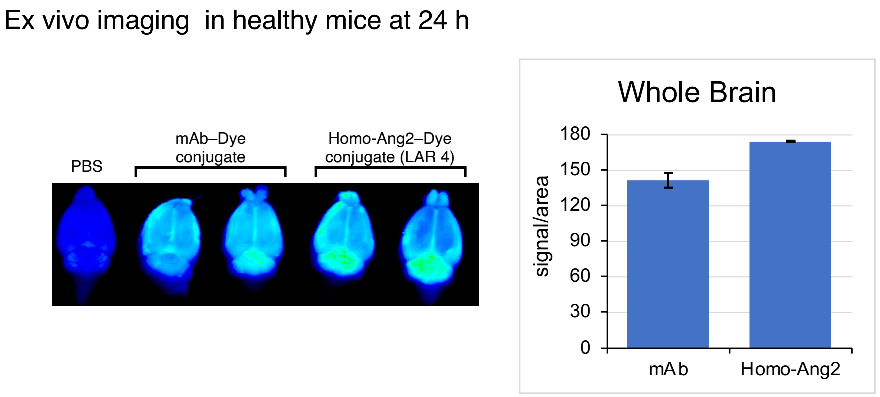

Fig. S3. Biodistribution study in non-tumor-bearing (healthy) mice. Ex vivo fluorescence images of whole brains harvested from CD-1 mice 24 hours after intravenous injection of each fluorescent conjugate at 3 mg/kg (n = 1 for PBS and n = 2 for each conjugate). Semi-quantification of the Cy5.5 signal in the whole brains.

Supplementary References

1 Y. Anami, W. Xiong, X. Gui, M. Deng, C. C. Zhang, N. Zhang, Z. An and K. Tsuchikama, *Org. Biomol. Chem.*, 2017, **15**, 5635–5642.

2 Y. Anami and K. Tsuchikama, *Methods Mol. Biol.*, 2020, **2078**, 71–82.

3 Y. Anami, C. M. Yamazaki, W. Xiong, X. Gui, N. Zhang, Z. An and K. Tsuchikama, *Nat. Commun.*, 2018, **9**, 2512.

4 Y. Anami, M. Deng, X. Gui, A. Yamaguchi, C. M. Yamazaki, N. Zhang, C. C. Zhang, Z. An and K. Tsuchikama, *Mol. Cancer Ther.*, 2020, **19**, 2330–2339.

5 C. M. Yamazaki, A. Yamaguchi, Y. Anami, W. Xiong, Y. Otani, J. Lee, N. T. Ueno, N. Zhang, Z. An and K. Tsuchikama, *Nat. Commun.*, 2021, **12**, 3528.
